# Awake' Extracorporeal Membrane Oxygenation Requires Adequate Lower Body Muscle Training and Mobilisation As Successful Bridge to Lung Transplant

**DOI:** 10.1186/2197-425X-3-S1-A510

**Published:** 2015-10-01

**Authors:** JAJM Hermens, SA Braithwaite, G Heijnen, D van Dijk, DW Donker

**Affiliations:** University Medical Center Utrecht, Department of Intensive Care Medicine, Utrecht, Netherlands; University Medical Center Utrecht, Department of Anesthesiology, Utrecht, Netherlands; University Medical Center Utrecht, Department of Physical Therapy, Utrecht, Netherlands

## Introduction

‘Awake’ extracorporeal membrane oxygenation (ECMO) is being used increasingly as a bridge to lung transplant (LuTx) to support refractory respiratory failure as an alternative to invasive mechanical ventilation and associated immobilisation and deconditioning which are associated with poor outcome. the ideal combination of ECMO cannulation and muscle training has yet to be determined and depends largely on patient-specific needs and procedural limitations.

## Methods

**Figure 1:**
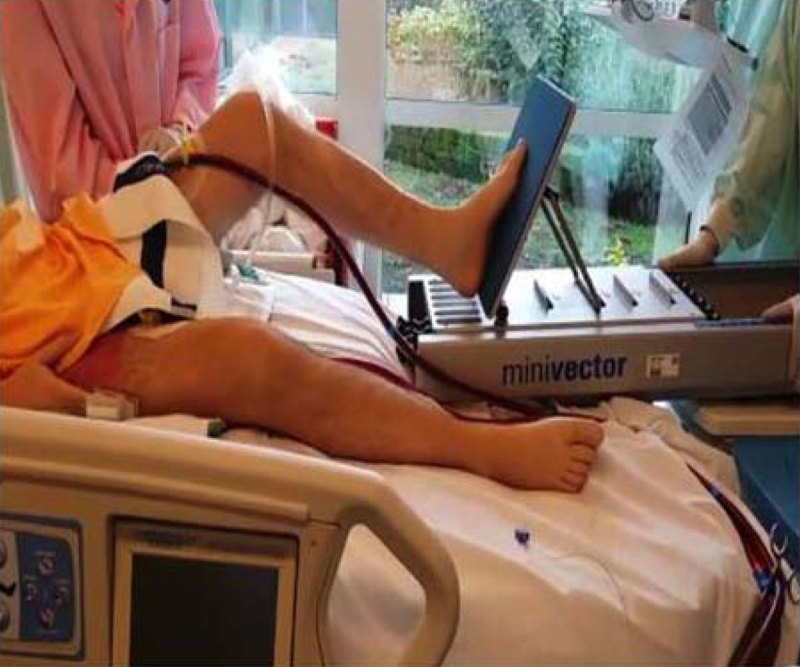
Figure 1

Retrospective analysis included all 'awake', non-intubated patients with end stage lung disease requiring ECMO as bridge to LuTx between 2008 and 2015. Veno-venous (VV) ECMO was initiated after informed consent and agreement within the multidisciplinary transplant team on a case-to-case basis. With as indication persistent hypoxemia (SaO2 < 80%) and/or refractory hypercapnia with acidosis (pH < 7.20) despite maximal non-invasive support. Immediate initiation of mobilisation was considered high priority in all patients and comprised: 1. extensive sputum mobilisation 2. muscle training of the lower extremities which entailed dynamic quadriceps training by leg press (Figure 1), bed bike or squats from sitting position and 3. bed-to-chair mobilisation. Muscle strength was evaluated semi-quantitatively before and during the mobilisation program and indicated on the 'Medical Research Council' (MRC) scale.

## Results

Nine patients (median age 35, 16-59 (range), 4 male) with end stage lung disease (7 cystic fibrosis, 1 idiopathic pulmonary fibrosis, 1 lymphangioleiomyomatosis) were supported with VV ECMO (mean duration 12 days, range: 5-19 days). Four were successfully bridged to LuTx, 5 patients did not survive to transplant. Three different cannulation modes were used including the Avalon Elite® bi-caval dual lumen catheter, femoral-jugular and bifemoral cannulation. in all surviving patients, mobilisation was successful and improved lower body muscle strength (mean MRC before training was 3.75 (range 3-4) and mean MRC 1 day pre-LuTx was 4.25 (range 4-5). of the nonsurviving patients, only one patient was physically able to perform muscle training but gained no improvement of muscle strength. Complications during mobilisation were only observed with bi-femoral cannulation and included one patient with a large rectus haematoma and a separate case with an obstructing thrombus in the return cannula.

## Conclusions

Mobilization and improvement of lower body muscle strength are prerequisites for successful bridging to LuTx with 'awake', non-intubated VV ECMO independent of cannulation technique.

## Consent to publish

Consent to publish the image in Figure 1 was obtained from the patient.

